# Effects of Porcine Immature Oocyte Vitrification on Actin Microfilament Distribution and Chromatin Integrity During Early Embryo Development *in vitro*

**DOI:** 10.3389/fcell.2021.636765

**Published:** 2021-04-19

**Authors:** Alma López, Yvonne Ducolomb, Eduardo Casas, Socorro Retana-Márquez, Miguel Betancourt, Fahiel Casillas

**Affiliations:** ^1^Biological and Health Sciences Program, Metropolitan Autonomous University-Iztapalapa, Mexico City, Mexico; ^2^Department of Health Sciences, Metropolitan Autonomous University-Iztapalapa, Mexico City, Mexico; ^3^Department of Biology of Reproduction, Metropolitan Autonomous University-Iztapalapa, Mexico City, Mexico

**Keywords:** vitrification, embryo development, immature oocytes, porcine (pig) model, actin microfilaments, chromatin, *in vitro* fertilization-embryos

## Abstract

Vitrification is mainly used to cryopreserve female gametes. This technique allows maintaining cell viability, functionality, and developmental potential at low temperatures into liquid nitrogen at −196°C. For this, the addition of cryoprotectant agents, which are substances that provide cell protection during cooling and warming, is required. However, they have been reported to be toxic, reducing oocyte viability, maturation, fertilization, and embryo development, possibly by altering cell cytoskeleton structure and chromatin. Previous studies have evaluated the effects of vitrification in the germinal vesicle, metaphase II oocytes, zygotes, and blastocysts, but the knowledge of its impact on their further embryo development is limited. Other studies have evaluated the role of actin microfilaments and chromatin, based on the fertilization and embryo development rates obtained, but not the direct evaluation of these structures in embryos produced from vitrified immature oocytes. Therefore, this study was designed to evaluate how the vitrification of porcine immature oocytes affects early embryo development by the evaluation of actin microfilament distribution and chromatin integrity. Results demonstrate that the damage generated by the vitrification of immature oocytes affects viability, maturation, and the distribution of actin microfilaments and chromatin integrity, observed in early embryos. Therefore, it is suggested that vitrification could affect oocyte repair mechanisms in those structures, being one of the mechanisms that explain the low embryo development rates after vitrification.

## Introduction

Currently, vitrification is mostly used to cryopreserve gametes and embryos. It is intended to maintain cell viability, functionality, and developmental potential when they are stored at low temperatures (Chian et al., 2004; Casillas et al., 2018). In recent years, vitrification has been a useful tool for assisted reproduction techniques (ART), so that the scientific and technical progress in this field has been developed for female gametes. In humans, it is considered an important resource in the treatment of reproductive conditions and infertility (Khalili et al., 2017), as well as to improve the reproductive capacity and gamete quality in economically important and endangered species (Mullen and Fahy, 2012). The cryoprotectant agents (CPAs) are substances that protect cells during cooling and warming. However, their use in high concentrations increases the risk of osmotic damage caused by their chemical components (Chian et al., 2004). Although substantial progress has been made to improve vitrification protocols by the use of co-culture systems (Jia et al., 2019), the Cryotech method (Angel et al., 2020; Nowak et al., 2020), the reduction of the volume of cryopreservation cell devices, and CPA selection (Sun et al., 2020), the recovery of intact morphophysiological gametes after vitrification are still low due to the damage generated in cell structures, mainly the plasma membrane, cytoplasm, nucleus, and DNA (Chang et al., 2019). In this regard, it was reported that the extent of the cell damage depends on the nuclear cell stage (Somfai et al., 2012; Egerszegi et al., 2013).

During vitrification, the addition of CPAs is required for cell protection, and it depends on the animal species, the cell type, and the chemical nature of the CPAs to select an appropriate vitrification procedure. In pigs, vitrification can cause alterations in actin microfilaments (MF) and chromatin (CHR), affecting oocyte viability, maturation, fertilization, and embryo development (ED). Previous studies have evaluated the effect of vitrification on germinal vesicle (GV), metaphase II (MII) oocytes, zygotes, and blastocysts in the same stage of development (Egerszegi et al., 2013). Other studies evaluated the role of MF and CHR based on the fertilization and ED rates, but they did not determine the alterations of these structures (Rajaei et al., 2005; Egerszegi et al., 2013). Therefore, this study was designed to evaluate how the vitrification of porcine immature oocytes affects early ED according to the distribution of actin MF and CHR integrity.

## Materials and Methods

### Ethics Statement and Animal Care

This study was approved under the regulations of the Ethics Committee for care and use of animals, Metropolitan Autonomous University-Iztapalapa Campus.

### Experimental Design

Five replicates were performed for all experiments. After selection, the cumulus–oocyte complexes (COCs) were divided into two groups: (a) control group, fresh GV oocytes underwent *in vitro* maturation (IVM), and subsequently fertilized *in vitro* (IVF) for early ED (two to four blastomeres) through 40 h and (five to eight blastomeres) through 68 h. (b) Experimental group, vitrified GV oocytes, then IVM in a co-culture with fresh granulosa cells, followed by IVF and early ED. In both groups, the viability in oocytes and embryos was evaluated by methyl tetrazolium (MTT) staining. IVM, IVF, and ED were evaluated by bisbenzimide (Hoechst 33342) staining. The analysis of actin MF distribution was carried out using phalloidin–fluorescein isothiocyanate conjugate (phalloidin–FITC), and CHR by Hoechst staining (Rojas et al., 2004).

### Chemicals, Culture Media, and Culture Conditions

Unless otherwise stated, all reagents were purchased from Sigma Chemical Co. (St. Louis, MO, United States), and different culture media were prepared in the laboratory. For COCs collection and washing, Tyrode’s medium containing 10 mM HEPES, 10 mM sodium lactate, and 1 mg/ml of polyvinyl alcohol (TL-HEPES-PVA) were used (Abeydeera et al., 1998).

For oocyte vitrification and warming, TCM-199-HEPES medium was supplemented with 0.5 mM L-glutamine and 0.1% 200 PVA (VW medium). To perform IVM, the maturation medium (MM) consisted of TCM 199 with Earle’s salt medium, supplemented with 26.2 mM sodium bicarbonate, 0.1% PVA, 3.05 mM D-glucose, 0.91 mM sodium pyruvate, 0.57 mM cysteine, and 10 ng/ml of EGF (In Vitro, Mexico).

The medium for fertilization was Tris-buffered (mTBM) containing 3 mM KCl, 13.1 mM NaCl, 7.5 mM CaCl_2_, 20 mM Tris, 11 mM glucose, and 5 mM sodium pyruvate, 0.4% fraction V bovine serum albumin (BSA), and 2.5 mM caffeine (Abeydeera and Day, 1997).

The medium for early embryo culture was North Carolina State University-23 (NCSU-23) medium supplemented with 0.4% BSA (Petters and Wells, 1993). All culture media and samples were incubated under mineral oil at 38.5°C with 5% CO_2_ in air and humidity at saturation.

### Oocyte Collection

Porcine ovaries were obtained from pre-pubertal Landrace gilts at “Los Arcos” slaughterhouse, Edo. de México and transported to the laboratory in 0.9% NaCl solution at 25°C. The slaughterhouse is registered in health federal law authorization under the number 6265375. For COCs collection, ovarian follicles between 3 and 6 mm in diameter were punctured using an 18-gauge needle set to a 10-ml syringe. Oocytes with intact cytoplasm and surrounded by cumulus cells were selected for the assays.

### Vitrification and Warming

For vitrification, COCs were washed twice in VW medium and equilibrated in the first vitrification solution containing 7.5% dimethylsulfoxide (DMSO) and 7.5% ethylene glycol (EG) for 3 min. Then, COCs were exposed to the second vitrification solution containing 16% DMSO, 16% EG, and 0.4 M sucrose for 1 min, and at least nine oocytes were immersed in a 2-μl drop and loaded into the Cryolock (Importadora Mexicana de Materiales para Reproducción Asistida S. A. de C.V. Mexico). Finally, in less than 1 min, the Cryolock was plunged horizontally into liquid nitrogen at −196°C, then COCs were vitrified for 30 min (Casillas et al., 2014). For warming, the one-step method was performed (Sánchez-Osorio et al., 2010). For COCs recovery, the Cryolock was immersed vertically in a four-well dish containing 800 μl of VW medium with 0.13 M sucrose. Immediately, oocytes were incubated in the same medium for 5 min, then recovered for IVM (Sánchez-Osorio et al., 2008).

### *In vitro* Maturation

Control and vitrified-warmed COCs were washed in 500 μl of MM three times. Afterward, 30–40 oocytes were randomly distributed in a four-well dish (Thermo-Scientific Nunc, Rochester NY) containing 500 μl of MM with 0.5 μg/ml of LH and 0.5 μg/ml of FSH (Ducolomb et al., 2009) for 44 h (Casas et al., 1999). Vitrified oocytes were matured in MM with a co-culture with fresh granulosa cells for 44 h (Casillas et al., 2014). The total numbers of evaluated cells in the control and vitrification groups were 256 and 143, respectively.

### *In vitro* Fertilization

After IVM, oocytes were denuded mechanically with a 100-μl micropipette. Then, the oocytes were washed three times in MM and three times in mTBM in 500-μl drops covered with mineral oil. For IVF, 40–60 oocytes were placed in 50-μl drops of mTBM covered with mineral oil and incubated at 38.5°C with 5% CO_2_ and humidity at saturation for 1 h until insemination (Ducolomb et al., 2009).

Semen was obtained by the gloved hand method on a commercial farm; a 1:10 dilution was made with boar semen extender (MR-A, Kubus, S.A.) and transported to the laboratory at 16°C. Five microliters of semen was diluted in 5 ml of PBS-Dulbecco, Gibco, 1:1 dilution, supplemented with 0.1% BSA fraction V, 0.1 μg/ml of potassium penicillin G, and 0.08 μg/ml of streptomycin sulfate. It was centrifuged at 61 × *g* at 25°C for 5 min. The supernatant was diluted 1:1 with PBS-Dulbecco and centrifuged at 1,900 × *g* for 5 min. The supernatant was removed and suspended in 10 ml of PBS-Dulbecco and centrifuged at 1,900 × *g* for 5 min. The pellet was suspended with 100 μl of mTBM. From this solution, 10 μl was diluted 1:1,000 with mTBM to calculate a final concentration of 5 × 10^5^ spermatozoa/ml. Finally, 50 μl of the sperm suspension was co-incubated for 6 h with the matured oocytes for fertilization at 38.5°C (Ducolomb et al., 2009). The fertilization rate was determined through pronuclei (PNs) formation, and the total numbers of evaluated cells in the control and vitrification groups were 126 and 95, respectively.

### Embryo Development

After co-incubation, oocytes were washed three times in 50-μl drops of NCSU-23 medium (Petters and Wells, 1993) supplemented with 0.4% fatty acid–free BSA and placed in drops of 500 μl of the same medium covered with mineral oil in a four-well dish and incubated for 16 h. The evaluation of early ED was performed after 40 and 68 h of incubation (Ducolomb et al., 2009). The total numbers of evaluated cells in the control and vitrification groups were 241 and 151, respectively.

### Evaluation of Oocytes and Embryo Viability

**FIGURE 1:**
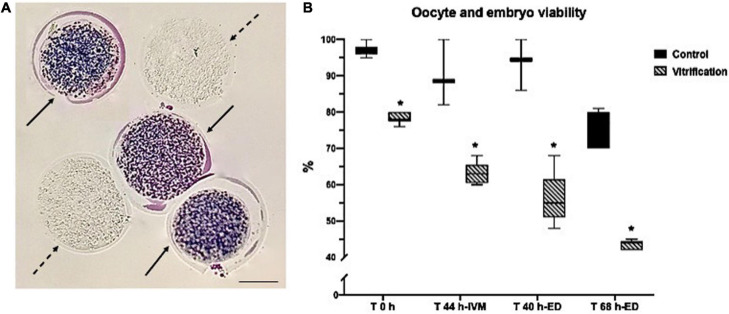
Oocyte and embryo viability evaluation by methyl tetrazolium (MTT) staining in control and vitrified groups: **(A)** representative image for oocyte viability evaluation criteria (continuous arrows show purple living cells, and dotted arrows show colorless dead cells); 10×, scale bar = 50 μm; **(B)** percentage of oocyte viability at T 0 h, after T 44 h-IVM and early embryos after T 40 and 68 h-ED; the total numbers of evaluated cells in the control and vitrification groups were 386 and 381, respectively. *Significant difference vs. control. *P* < 0.05.

Viability was analyzed in oocytes and embryos with MTT staining at T 0 h after oocyte collection, and vitrification, after 44 h of IVM, and after 40 and 68 h of early ED (Mosmann, 1983). Oocytes and embryos were stained with 100-μl drops of 0.5 mg/ml of MTT diluted in mTBM. After 1.30 h, oocytes and embryos were observed under a light microscope (Zeiss Axiostar). Cells showing a purple stain were considered alive, and those colorless were considered dead (Figure 1A). The total numbers of evaluated cells in the control and vitrification groups were 386 and 381, respectively.

### Evaluation of Oocyte Maturation

**FIGURE 2:**
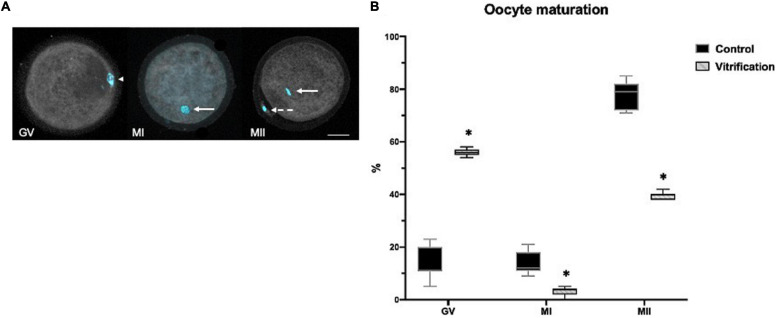
Oocyte *in vitro* maturation (IVM) evaluation by Hoechst staining in control and vitrified groups: **(A)** representative image for oocyte IVM evaluation criteria (oocytes were classified as: arrowhead shows germinal vesicle (GV), continuous arrow shows metaphase I (MI), continuous arrow shows metaphase II (MII), and the dotted arrow show the polar body; 400×, scale bar = 30 μm; **(B)** percentage of oocyte IVM; the total numbers of evaluated cells in the control and vitrification groups were 256 and 143, respectively. ^∗^Significant difference vs. control. *P* < 0.05.

Maturation was evaluated by Hoechst stain. Oocytes were stained with 10 μg/ml of Hoechst for 40 min using a confocal scanning laser microscope (Zeiss, LSM T-PMT) for observation. Maturation was evaluated at 44 h of incubation; oocytes with a germinal vesicle (GV) or in metaphase I (MI) were considered as immature and those in metaphase II (MII) with the first polar body as mature (Figure 2A).

### Evaluation of *in vitro* Fertilization

**FIGURE 3:**
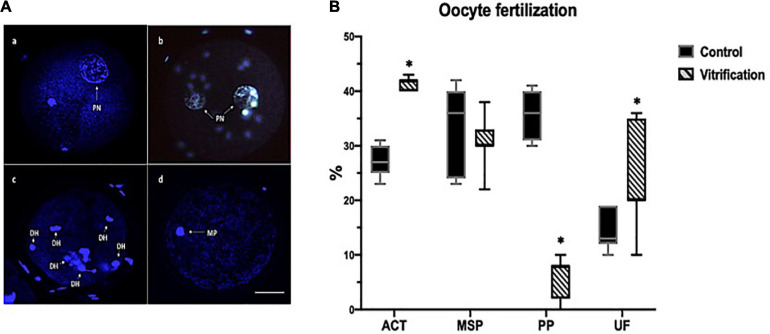
Oocyte *in vitro* fertilization (IVF) evaluation by Hoechst staining in the control and vitrified groups. **(A)** Representative images for IVF evaluation criteria: (a) activated oocyte, arrow shows one PN; (b) monospermic zygotes, arrows show two PN; (c) polyspermic oocyte, arrows show decondensed sperm heads, and (d) unfertilized oocytes, arrow shows the metaphase I; pronuclei (PN), decondensed sperm heads (DH), metaphase (MP); **(B)** percentage of oocyte IVF; activated (ACT), monospermic (MSP), polyspermic (PP), and unfertilized oocytes (UF); the total numbers of evaluated cells in the control and vitrification groups were 126 and 95, respectively; 200×, scale bar = 30 μm. ^∗^Significant difference vs. control. *P* < 0.05.

Zygotes and embryos were stained with 10 μg/ml of Hoechst for 40 min using Zeiss, LSM T-PMT for observation. To evaluate fertilization, oocytes with one pronucleus (PN) were considered activated (ACT) (Figure 3Aa) and those with two pronuclei as monospermic (MSP) (Figure 3Ab), and more than two decondensed sperm heads (DH) or more than two-pronuclei were considered polyspermic (PP) (Figure 3Ac). Oocytes in MII (first polar body) were considered as not fertilized (UF) (Figure 3Ad).

### Evaluation of Embryo Development

**FIGURE 4:**
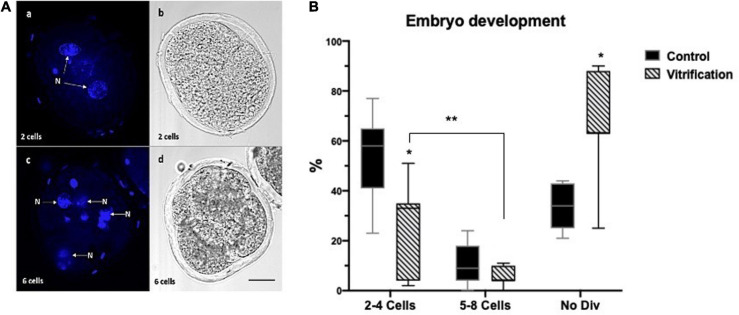
Early embryo development (ED) evaluation by Hoechst staining in the control and vitrified groups. **(A)** Representative images for early ED evaluation criteria; (a,c) blastomeres stained with Hoechst; (b,d) clear field showing embryos blastomeres; (N) nucleus; the total numbers of evaluated cells in the control and vitrification groups were 241 and 151, respectively; 200×, scale bar = 30 μm; **(B)** percentage of early ED [two to four cells, five to eight cells, and non-divided (No Div)]; *Significant difference vs. control. *P* < 0.05. **Significant differences between groups. *P* < *0.05.*

Early ED was evaluated 40 h after IVF, two to four cell embryos (Figure 4Aa), and 68 h after IVF five to eight cell embryos (Figure 4Ac).

### Hoechst Staining and Immunocytochemistry (Actin Microfilaments and Chromatin)

For CHR evaluation, embryos were stained with Hoechst and, for actin MF, by immunofluorescence with phalloidin-FITC, 1:350 in PBS. After 40 and 68 h of incubation, early embryos were washed three times to 500-μl drops of PBS-BSA; then, 300 μl of Hoechst was added and kept at 4°C for 45 min; afterward, 200 μl of 4% paraformaldehyde fixative solution was added and kept at 4°C overnight. After, 200 μl of PBS–Triton X-100 1% permeabilizing solution was added at 4°C for 2 h and washed. Next, 200 μl of blocking solution was added, with 0.02 g/ml of PBS–BSA, 0.02 g/ml of skimmed milk, and 0.011 g/ml of glycine diluted in PBS, for 1 h at room temperature. For MF labeling, 200 μl of the phalloidin–FITC was added and kept at 4°C for 2 h, then, transferred three times to 500-μl drops. All the incubations were performed in the dark. The washing of the embryos was made with PBS–BSA. The slide mounting was performed with PBS/glycerol 1:9 on slides and covered with a coverslip and sealed with transparent nail polish (Rojas et al., 2004).

Images were obtained using Zeiss, LSM T-PMT. The analysis was carried out capturing Z stack series, through four sections covering the whole embryo. MF visualization (green) was by Phalloidin–FITC with an excitation wavelength of 490 nm and an emission of 525 nm. For CHR (blue), Hoechst had an excitation of 350 nm and an emission of 470 nm. The evaluation of images was performed using the Image J Processor.

**FIGURE 5:**
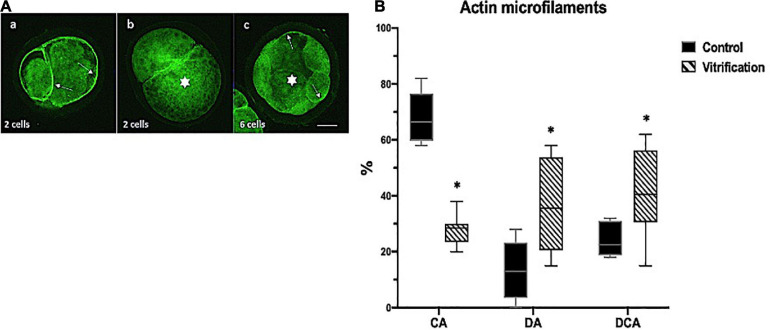
Evaluation of actin microfilaments (MF) in early embryos by phalloidin-fluorescein isothiocyanate (FITC) staining in the control and vitrified groups; **(A)** representative images for actin microfilament evaluation criteria in early ED: (a) white arrows show cortical actin (CA); (b) white star shows dispersed actin (DA), and (c) white star shows dispersed cortical actin (DCA); the total numbers of evaluated cells in the control and vitrification groups were 61 and 35, respectively; 200×, scale bar = 30 μm; **(B)** percentage of actin microfilaments in early ED. ^∗^Indicate significant difference vs. control. *P* < 0.05.

**FIGURE 6:**
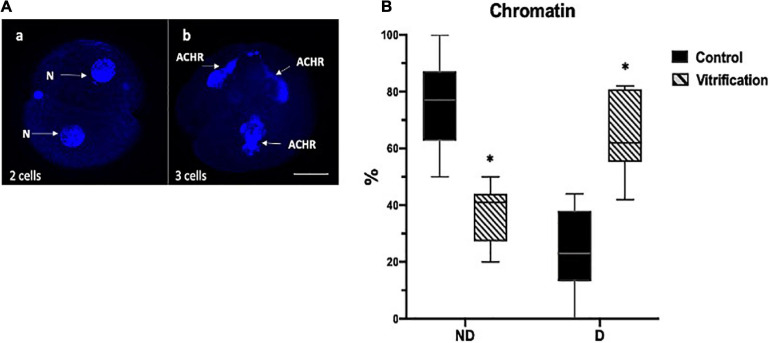
Chromatin (CHR) evaluation in early ED by Hoechst staining in control and vitrified groups. **(A)** representative images for CHR evaluation criteria in early ED; (a) 2-Cell embryo, white arrows show nucleus without damage (ND), and (b) 3-Cell embryo, white arrows show abnormal chromatin (ACHR); the total numbers of evaluated cells in the control and vitrification groups were 64 and 33, respectively; 200×, scale bar = 30 μm; **(B)** percentage of CHR in early ED. ^∗^Significant difference vs. control. *P* < 0.05.

For actin MF distribution evaluation, embryos were classified as embryos with cortical actin (CA) (Figure 5Aa), disperse actin (DA) (Figure 5Ab), and dispersed cortical actin (DCA) (Figure 5Ac). Embryos showing CA were considered with good quality and high developmental potential (Figure 5Aa); DCA was considered as a medium quality embryo indicator (Figure 5Ac) and DA as a low embryo quality, with less developmental potential (Figure 5Ab). For CHR evaluation, two classifications in both groups were considered: embryos without damage (ND) and with damage (D) (Figure 6A). The ND CHR embryos presented well-defined nuclei (Figure 6Aa). D CHR embryos were considered when one or more abnormal chromatin (ACHR) structures were identified (Figure 6Ab). ND CHR embryos are related to good quality with a high probability of successful ED. D CHR embryos have less embryo development potential. For MF evaluation, the total numbers of evaluated cells in the control and vitrification groups were 61 and 35, respectively. For CHR evaluation, the total numbers of evaluated cells in the control and vitrification groups were 64 and 33, respectively.

### Statistical Analysis

Statistical analyses were carried out using GraphPad Prism 8.2.1 (Graphpad Software Inc.). Data from vitrified and control groups were compared with non-parametric Mann–Whitney *U* test with a confidence level of *P* < 0.05, and percentage data are presented as mean ± standard deviation (SD) values.

## Results

### Evaluation of Oocytes, Embryo Viability, and Oocyte Maturation

After collection (T 0 h), 97% of the control oocytes were alive, but this percentage was reduced significantly (78%) after vitrification (*P* < 0.05). Oocyte viability after 44 h of *in vitro* maturation in the control was 89%, while in the vitrified group, it was 63% (*P* < 0.05). After 40 h of embryo development, 94% of embryos were alive in the control and decreased significantly in the vitrified group up to 54% (*P* < 0.05). After 68 h of embryo development, viability decreased up to 42% (*P* < 0.05) in the vitrification group compared with that of the control (Figure 1B).

Figure 2B shows the percentage of oocytes *in vitro* maturation in both groups. Maturation (metaphase II-first polar body) was significantly lower in the vitrification group 40% compared with the control 79% (*P* < 0.05). A higher germinal vesicle rate (58%) was obtained in the vitrification group (*P* < 0.05) compared with that of the control (12%). Also, the percentage of metaphase I oocytes was significantly lower in the vitrification group (*P* < 0.05) compared with that of the control (4 and 12%, respectively).

### Evaluation of *in vitro* Fertilization and Embryo Development

*In vitro* fertilization results indicated that vitrified oocytes displayed a higher percentage (*P* < 0.05) of activated (one-pronucleus) and unfertilized (without pronuclei formation) rates compared with that of the control (42 vs. 27%, 20 vs. 13%, respectively); however, polyspermic fertilization (more than two-pronuclei) in vitrified oocytes was significantly lower (*P* < 0.05) than that of the control (8 vs. 36%, respectively). Meanwhile, monospermic fertilization (two-pronuclei) had no significant difference between both groups (*P* > 0.05) (36% control vs. 31% vitrified) (Figure 3B).

The percentage of early embryo development at 40 and 68 h of incubation in both groups are shown in Figure 4B. In the control group, a higher percentage of embryos with two to four cells (58%) was found compared with the vitrification group (33%) (*P* < 0.05). In vitrified oocytes, a higher percentage of undivided embryos (No Div) (63%) was obtained compared with that in the control (33%) (*P* < 0.05). However, both groups were not statistically different in the production of five to eight cell embryos (*P* > 0.05) (9% control vs. 4% vitrified). Also, the percentage of embryos that reached five to eight cells was significantly lower after vitrification than the two to four cells (*P* > 0.05).

### Evaluation of Actin Microfilament Distribution and Chromatin Integrity in Early Embryos

Results in the control group showed a higher percentage of embryos with cortical actin compared with the vitrification group (*P* < 0.05) (67 vs. 29%). In the vitrified group, higher percentages of dispersed actin (32 vs. 11%) and dispersed cortical actin (40 vs. 23%) were obtained compared with the control (*P* < 0.05) (Figure 5B).

For chromatin evaluation, results indicate that in the control, a higher percentage of embryos with chromatin without damage, with well-defined nuclei was obtained compared with the vitrification group (*P* < 0.05) (77 and 41%, respectively). Also, there was a greater percentage of embryos in the vitrification group with chromatin damage, with one or more scattered chromatin structures and without embryo division (61 and 23%, respectively) (Figure 6B).

**FIGURE 7:**
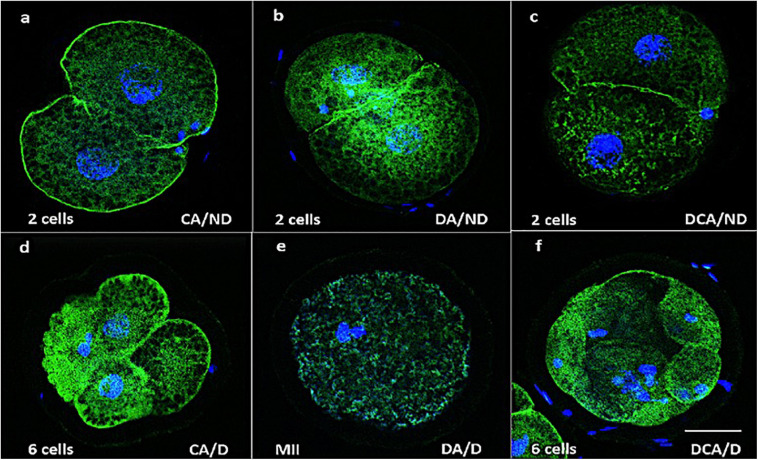
Representative merged images of actin microfilaments (green) and chromatin (blue) in early ED. **(a–c)** Images correspond to embryos from fresh oocytes, and **(d–f)** images correspond to embryos from vitrified oocytes. **(a)** Cortical actin without damage (CA/ND); **(b)** dispersed actin without damage (DA/ND); **(c)** dispersed cortical actin without damage (DCA/ND); **(d)** cortical actin with damage (CA/D); **(e)** dispersed actin with damage (DA/D); **(f)** dispersed cortical actin with damage (DCA/D); 200×, scale bar = 30 μm.

Figure 7 shows the merged images from microfilaments and chromatin evaluation in early embryos. Pictures a, b, and c correspond to the control group and images d, e, and f to the vitrification group. Some embryos show damaged blastomeres, and others did not divide. It seems that the cortical distribution of actin microfilaments and the integrity of the chromatin are related to embryo quality. Oocytes with dispersed actin also showed abnormal chromatin distribution and even the absence of cell division (Figure 7a). In contrast, control embryos showed normal chromatin conformation (ND), with cortical actin, or with some degree of actin dispersion (Figures 7a,c).

## Discussion

Over the past years, several methods for cryopreservation have been developed through the vitrification of immature (Casillas et al., 2018) and mature oocytes (Casillas et al., 2020) as well as for embryos in different developmental stages. In general, studies have shown that embryos have greater probabilities of survival after vitrification than immature and mature oocytes.

Immature oocyte vitrification in women is important because ovarian hyperstimulation can be avoided. Also, in other mammalian species, it is possible to recover a greater number of GV oocytes than MII. It has been reported that vitrification success in immature oocytes depends on the species and so different strategies are used. To evaluate the quality of embryos produced *in vitro* from vitrified immature oocytes, several aspects must be considered through the oocyte maturation, fertilization, as well as ED, like CPAs, containers, warming procedures, and recently the use of cumulus cell–oocyte co-culture (Casillas et al., 2015; Kopeika et al., 2015; de Munck and Vajta, 2017).

Although there is great progress in the knowledge of oocyte vitrification, the rate of embryos reaching morulae and blastocyst stages remains low; therefore, a few births of live offspring from vitrified immature oocytes are reported (Somfai et al., 2010). Several studies have used different approaches to explain the possible causes for the decrease in viable embryos produced from vitrified oocytes. One of the main parameters affected by vitrification is oocyte viability. After 44 h of IVM, in the vitrification group, it was 63% lower than that of the control group, which was 89% (Casillas et al., 2014). According to the literature, this is the first study that directly evaluates the viability of embryos derived from vitrified immature oocytes. In several species, studies reported only the percentage of ED to consider this parameter (Rajaei et al., 2005; Somfai et al., 2010; Chatzimeletiou et al., 2012; Fernández-Reyes et al., 2012). In the present study, results indicate that viability decreased significantly in embryos derived from vitrified oocytes compared with that of the control group (54 vs. 94%, respectively). This indicates that vitrification affects oocyte differentiation and ED *in vitro*. Although oocytes survived during this process, they were affected to carry out an optimal ED. However, the percentage of fertilized oocytes was similar in both groups. The percentage of polyspermy was lower in vitrified oocytes compared to the control.

In this study, there was a decrease in the IVM rate in vitrified oocytes compared with the control group. Similar results were previously reported (Casillas et al., 2020). According to the literature, studies evaluating IVM in vitrified oocytes have reported different results. This may be due to the oocyte maturation stage before vitrification (GV), cell containers, types of cryoprotectants, temperatures, or different cooling and warming procedures (Fernández-Reyes et al., 2012; Casillas et al., 2014; Wu et al., 2017). In addition, Somfai et al. (2010) reported 77% of IVM in the control vs. 22% in the vitrified oocytes using the solid surface vitrification method. These results are similar to those obtained in the present study; however, they use maturation media supplemented with porcine follicular fluid.

Actin is an essential component of the cytoskeleton that achieves functions such as cell migration and division, and the regulation of gene expression, which are basic processes for ED. Besides, microfilaments rearrange the organelles involved in fertilization (Sun and Schatten, 2006), such as extrusion of the second polar body and reorganization of the smooth endoplasmic reticulum for the generation of intracellular Ca^2+^ during oocyte activation (Chankitisakul et al., 2010; Bunnell et al., 2011; Gualtieri et al., 2011; Egerszegi et al., 2013; Martin et al., 2017). In mammals, the cortical distribution of actin microfilaments in mature oocytes is polarized, which is evidenced by swelling near the metaphase axis and less in the opposite cortical domain. In immature oocytes, the actin microfilament distribution does not appear polarized. This indicates that F-actin has a restructuration for polymerization during oocyte maturation (Coticchio et al., 2014, 2015b). Some studies have evaluated the effect of oocyte vitrification in the cytoskeleton, showing an interruption in the cortical microfilaments network, as well as disorganization of the microtubule spindle, which led to chromosomal dispersion (Rojas et al., 2004; Chatzimeletiou et al., 2012).

These data could explain the possible mechanism of damage derived from immature oocyte vitrification. Our results showed significant differences in the percentage of early embryos with actin dispersion. Vitrification inhibits the correct polymerization of G-actin, reflected in an MF breakdown in the cytoplasm and its disruption in the cortical area (Baarlink et al., 2017; Misu et al., 2017).

In the vitrification group, there was a high rate of No Div embryos. The percentage of embryos that reached five to eight cells was also lower after vitrification than the two to four cells. This could be related to the lack of damage repair mechanisms in the oocytes. Some blastomeres in the early embryos showed lower cortical actin compared with that of the control group. Besides, some disrupted actin polymerization (dispersed actin and dispersed cortical actin). An increase in blastomere DNA fragmentation in blastocyst is attributed to the cryoprotectants (Rajaei et al., 2005). Also, it was reported that the cytotoxicity of these agents occurs in a greater proportion of cells with high metabolic activity as immature oocytes or embryos (Lawson et al., 2011). Cryoprotectants interrupt the cortical microfilament network, causing spindle depolymerization and disorganization, which leads to chromosomal dispersion that may trigger aneuploidies during the ED (Rojas et al., 2004; Chatzimeletiou et al., 2012).

Actin microfilaments also are involved in the immobilization of mitochondria to the cell cortex or sites with high ATP utilization (Boldogh and Pon, 2006). Depolarization and disorganization of the cytoskeleton will prevent the externalization of the meiotic organization toward the cortical zone and the alignment of the chromosomes on the equatorial axis in the oocytes (Gualtieri et al., 2011).

DNA is susceptible to a variety of chemical compounds and physical agents causing alterations in its conformation as a result of errors produced during replication, recombination, and repair (Coticchio et al., 2015a). Changes in chromatin are a result of histones modifying enzymes, which alter its post-transcription activities and ATP-dependent chromatin remodeling complexes (Farrants, 2008). Reduced production of ATP in human vitrified oocytes can be associated with depolymerization of actin microfilaments, causing failure in the DNA repair system, leading to chromatin disorders (Manipalviratn et al., 2011). In the present study, this mechanism may explain the high proportion of embryos derived from vitrified oocytes showing some type of damage in the chromatin affecting the DNA repair system.

It is important to highlight that several studies reported in the literature have evaluated the effect of vitrification in GV and MII oocytes, zygotes, and blastocysts at the same stages of development in which they were vitrified. In this study, the effect of vitrification was evaluated in the further early ED. Previous studies in zygotes or blastocysts analyzed only the role of actin microfilaments and chromatin in the success of the fertilization and embryo production (Wu et al., 2006; Somfai et al., 2010; Egerszegi et al., 2013); however, the distribution and the morphological characteristics of these structures in early ED blastomeres has not been evaluated. Therefore, the present study provides important information that reveals the damage caused by vitrification in immature oocytes and their further early ED.

## Conclusion

In conclusion, the results of this study indicate that the damage generated by the vitrification of immature oocytes affects viability, maturation, and the distribution of actin MF and CHR integrity, as observed in early embryos.

## Data Availability Statement

The raw data supporting the conclusions of this article will be made available by the authors, without undue reservation.

## Ethics Statement

This study was approved under the regulations of the Ethics Committee for care and use of animals; Metropolitan Autonomous University-Iztapalapa Campus.

## Author Contributions

AL developed the methodology, performed the experiments, analyzed the results, conducted the investigation, and prepared and wrote the original draft. YD also developed the methodology, performed the experiments, and analyzed the results. EC analyzed the results and reviewed and edited the manuscript. SR-M reviewed and edited the manuscript. MB and FC conceptualized the study and developed the methodology, software, data curation, prepared and wrote the original draft, conducted the visualization, investigation, supervision, validation of the study, reviewed, edited, and wrote the final manuscript. All authors contributed to the article and approved the submitted version.

## Conflict of Interest

The authors declare that the research was conducted in the absence of any commercial or financial relationships that could be construed as a potential conflict of interest.

## Abbreviations

ACHR: abnormal chromatin
ACT: activated
ART: assisted reproduction techniques
CHR: chromatin
CA: cortical actin
CA/D: cortical actin with damage
CA/ND: cortical actin without damage
CPAs: cryoprotectant agents
COCs: cumulus-oocyte complexes
DH: decondensed sperm heads
DA: dispersed actin
DA/D: dispersed actin with damage
DA/ND: dispersed actin without damage
DCA: dispersed cortical actin
DCA/D: dispersed cortical actin with damage
DCA/ND: dispersed cortical actin without damage
ED: embryo development
FITC: fluorescein isothiocyanate
GV: germinal vesicle
IVF: *in vitro* fertilization
IVM: *in vitro* maturation
MM: maturation medium
MI: metaphase I
MII: metaphase II
MTT: methyl tetrazolium
MF: microfilaments
MSP: monospermic
N: nucleus
ND: nucleus without damage
PP: polyspermic
PN: pronuclei
mTBM: tris-buffer medium
UF: unfertilized
VW: vitrification warming.
